# Impact of Reactive Species on Amino Acids—Biological Relevance in Proteins and Induced Pathologies

**DOI:** 10.3390/ijms232214049

**Published:** 2022-11-14

**Authors:** Celia María Curieses Andrés, José Manuel Pérez de la Lastra, Celia Andrés Juan, Francisco J. Plou, Eduardo Pérez-Lebeña

**Affiliations:** 1 Hospital Clínico Universitario of Valladolid, Avenida de Ramón y Cajal, 3, 47003 Valladolid, Spain; 2 Institute of Natural Products and Agrobiology, CSIC-Spanish Research Council, Avda. Astrofísico Fco. Sánchez, 3, 38206 La Laguna, Spain; 3 Cinquima Institute and Department of Organic Chemistry, Faculty of Sciences, Valladolid University, Paseo de Belén, 7, 47011 Valladolid, Spain; 4 Institute of Catalysis and Petrochemistry, CSIC-Spanish Research Council, 28049 Madrid, Spain; 5 Sistemas de Biotecnología y Recursos Naturales, 47625 Valladolid, Spain

**Keywords:** reactive species, ROS, RNS, RHS, amino acids, reactive stress, proteins, pathologies

## Abstract

This review examines the impact of reactive species RS (of oxygen ROS, nitrogen RNS and halogens RHS) on various amino acids, analyzed from a reactive point of view of how during these reactions, the molecules are hydroxylated, nitrated, or halogenated such that they can lose their capacity to form part of the proteins or peptides, and can lose their function. The reactions of the RS with several amino acids are described, and an attempt was made to review and explain the chemical mechanisms of the formation of the hydroxylated, nitrated, and halogenated derivatives. One aim of this work is to provide a theoretical analysis of the amino acids and derivatives compounds in the possible positions. Tyrosine, methionine, cysteine, and tryptophan can react with the harmful peroxynitrite or ^•^OH and ^•^NO_2_ radicals and glycine, serine, alanine, valine, arginine, lysine, tyrosine, histidine, cysteine, methionine, cystine, tryptophan, glutamine and asparagine can react with hypochlorous acid HOCl. These theoretical results may help to explain the loss of function of proteins subjected to these three types of reactive stresses. We hope that this work can help to assess the potential damage that reactive species can cause to free amino acids or the corresponding residues when they are part of peptides and proteins.

## 1. Introduction

**Figure 1:**
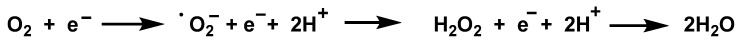
Reduction process of O_2_ to H_2_O, in several steps.

Reactive oxygen species ROS can be triggered by exogenous sources (tobacco, pollution, xenobiotics, drugs, ionizing radiation, etc.), but they can also be generated inside the cell by two different mechanisms: enzymatic and non-enzymatic; in both cases, they can have irreversible effects on animal and plant cells and tissues. The superoxide anion ^•^O_2_^−^ is unstable and cannot pass through membranes, but is rapidly converted to hydrogen peroxide H_2_O_2_ [1] and it is membrane-permeable. In the Fenton reaction, H_2_O_2_ produces the hydroxyl radical ^•^OH + ^−^OH, which is highly reactive in the mitochondrial matrix. Elevated levels of ROS lead to increased mtDNA damage [2]. The Gibbs free energy of O_2_ reduction process is negative, so it occurs spontaneously, seen in Figure 1. The Gibbs free energy is used to calculate the maximum amount of work that can be done by a thermodynamically closed system, with temperature and pressure being constant, and is a necessary condition in processes such as chemical reactions.

Mitochondria are an important source of ROS within most mammalian cells. The generation of mitochondrial ROS mainly takes place at the electron transport chain located on the inner mitochondrial membrane during the process of oxidative phosphorylation. Leakage of electrons at complex I and complex III from electron transport chains leads to partial reduction of oxygen to form ^•^O_2_^−^. Subsequently, ^•^O_2_^−^ is quickly dismutated to H_2_O_2_ by two superoxide dismutases: SOD(Mn) or SOD2 in the mitochondrial matrix, and SOD(Cu-Zn) or SOD1 into the inter-membrane space. Collectively, both ^•^O_2_^−^ and H_2_O_2_ generated are considered as mitochondrial ROS.

**Figure 2:**
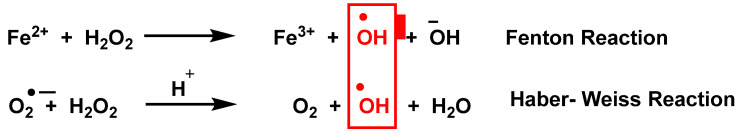
Production of ^•^OH by the Haber Weiss and Fenton reactions.

In addition to mitochondria, ROS are produced by a variety of enzymes such as NADPH oxidases NOXs, xanthine oxidase, nitric oxide synthase NOS, and in other cell organelles such as the endoplasmic reticulum, peroxisomes, and cytosol [3]. Haber Weiss and Fenton reactions [4] describe the production of ^•^OH, Figure 2.

Nitric oxide ^•^NO is a widely distributed biological signaling molecule found in a number of cell types, including macrophages, platelets, vascular endothelia, and neuronal cells [5]. In the cardiovascular system, ^•^NO is involved in myocardial contractility, inhibition of platelet aggregation, and limiting endothelial adhesion of leukocytes, thus participating in the etiology of cardiovascular diseases, such as hypertension, atherosclerosis, and myocardial depression associated with sepsis and septic shock, as well as reperfusion injury [6]. Vascular endothelial cells continuously produce ^•^NO, which modulates vascular tone. ^•^NO is produced from the nitrogen in the guanidine group of L-arginine when the terminal guanidine nitrogen atoms are oxidized [7] via a process catalyzed by the enzyme NO synthase NOS.

**Figure 3:**
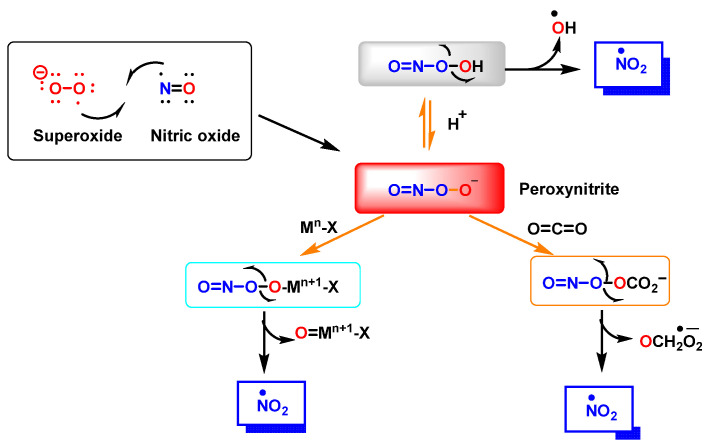
Reaction between ^•^NO and superoxide radicals ^•^O_2_^−^ produces peroxynitrite ONOO^−^. Secondary reactions of peroxynitrite to ^•^NO_2_, ^•^OH and ^•^CO_3_^−^. M^n^-X represents a metalloprotein, in which the co-ordinate metal moves up one valence to M^n+1^-X, as is the case for the superoxide dismutase SOD1 and SOD2. In the three secondary reactions of peroxynitrite ^•^NO_2_ is formed.

Peroxynitrite ONOO^−^ is produced by a reaction between ^•^NO and ^•^O_2_^−^, Figure 3. It is a potent oxidizing and nitrating agent with a relatively short half-life of about 10^−2^ s [8]. Its byproducts cause a variety of oxidative stress reactions, including lipid peroxidation, enzyme and protein inactivation, and mitochondrial dysfunction. Macrophages and other immune cells rely on the highly reactive chemical ONOO^−^ to clear invading pathogens [9]. Deregulation of ONOO^−^ production has been associated with an increased risk of cardiovascular disease, neurological disorders, and cancer [10]. Secondary reactions of peroxynitrite decomposition are synthesized in Figure 3.

Reactive nitrogen species (RNS) are detrimental to cellular function because the chemical changes they mediate can cause nitration, which in turn can affect the structures of cellular proteins, DNA, and lipids, impeding their normal function [11]. By interacting with tyrosine residues, ^•^NO_2_ can modify proteins and cause changes in protein function through nitration. The biological relevance of these modifications is underlined because they cause protein aggregation, turnover, signaling and immunological processes [12].

**Figure 4:**
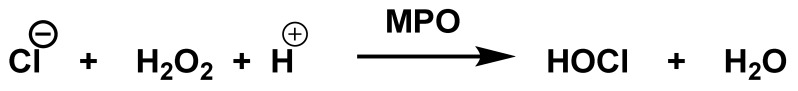
MPO-catalysed conversion of H_2_O_2_ and chloride to HOCl.

The enzyme myeloperoxidase MPO, also called hydrogen peroxide oxidoreductase, is present in macrophages, in different biological fluids (saliva, synovial fluid, and semen, among others) and in different tissues (heart, kidney, skin, liver, and placenta). However, the most common sources are neutrophils, where the enzyme is located at the lysosomal level, in the azurophil granules [13]. The main reaction catalyzed by MPO, under physiological conditions, is the oxidation of the Cl^−^ anion by H_2_O_2_ to give hypochlorous acid HOCl, a very reactive oxidizing agent, which can also act as a chlorinating agent and is the main strong oxidant generated by neutrophils in appreciable quantities [14]. MPO is the only peroxidase that catalyses the conversion of H_2_O_2_ and chloride to produce hypochlorous acid HOCl, Figure 4.

**Figure 5:**
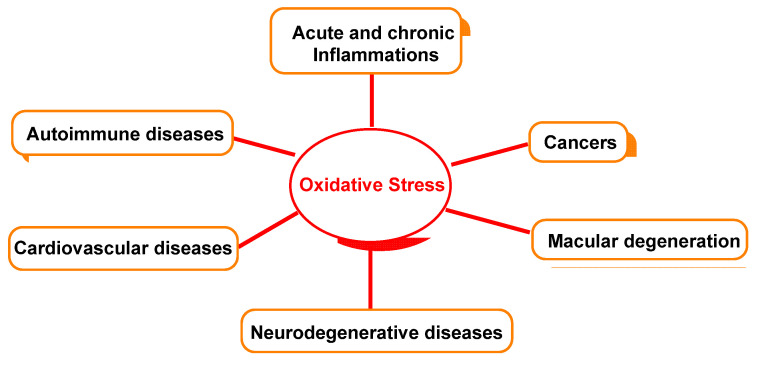
Oxidative-stress-relevant disease.

Figure 5 outlines the relationship between oxidative damage and possible associated diseases.

## 2. Reactive Stress on Amino Acids

In recent decades, there has been considerable interest in the idea that chronic oxidative/nitrosative stress plays a key role in the etiology of human disease [15]. These reactions, mediated by reactive oxygen species ROS and reactive nitrogen species RNS, are detrimental to cellular function, but do not present detectable disease-triggering symptoms [16]. Enhanced protection systems against oxygen and nitrogen radicals are thought to play a key role in primate evolution, resulting in increased longevity and lower rates of age-related diseases [17].

Peroxynitrite ONOO^−^ is an oxidant and nitrating agent that can penetrate biological membranes [18]. Peroxynitrite reacts with proteins through three possible pathways: (i) peroxynitrite reacts directly with cysteine, methionine, and tryptophan; (ii) peroxynitrite reacts rapidly with transition metal centers and selenium-containing amino acids; and (iii) free radicals formed during homolysis of peroxynitrite, such as hydroxyl and nitrogen dioxide radicals, and the carbonate radical ^•^CO_3_^−^ formed in the presence of carbon dioxide, also react with proteins. ^•^CO_3_^−^ is negatively charged in all physiological environments, including those of acidic pH such as the phagolysosomes of phagocytic cells and ischemic tissues. Although less oxidizing than the hydroxyl radical (E^0^ = 2.3 V, pH 7.0), the carbonate radical (E^0^ = 1.78 V, pH 7.0) is a very strong one-electron oxidant that acts by both electron transfer and hydrogen abstraction mechanisms to produce radicals from the oxidized targets. The inability of the carbonate radical to produce stable adducts makes it difficult to prove its production under physiological conditions. In contrast, other radicals, such as the hydroxyl radical and nitrogen dioxide, produce stable adducts with exogenous targets and biomolecules.

As a result, the changes caused by these radicals are more complex than those caused by hydroxyl radicals alone, since they lead to oxidation and nitration.

In vivo, protein nitration at the tyrosine residue is considered a biomarker for nitrosative stress. The oxidative stress biomarker, 3-nitrotyrosine, is useful for identifying major neurodegenerative diseases [19]. In this reaction, nitrotyrosine units are generated in peptides and proteins by a radical reaction. The nitration of tyrosine begins with the formation of the tyrosyl radical, which is formed when tyrosine is oxidized by the hydroxyl or carbonate radical. Following the mechanism of nitration of tyrosine already documented in the literature, the chemical mechanism of hydroxylation starts with the formation of the phenoxyl radical in the aromatic ring, just as in the nitration of tyrosine.

Although the vast majority of research on RNS-derived protein modifications has long focused on the nitration of Tyr residues, there is increasing evidence that nitrated or hydroxylated tryptophan may also play an important role in controlling cellular processes. Tryptophan bound to proteins is susceptible to modification by nitrating agents, and it is important to determine the nature of these modifications. This is because the nitration and oxidation products can take different forms. Yamakura et al. (2005) found that ONOO induced Trp modifications in human Cu, Zn-SOD, including 5- and 6-NO_2_-Trp, and kynurenine, oxindole-3-alanine, and dihydroxy-tryptophan [20]. The 2007 study by Ishii et al. showed that ONOO^−^ treatment leads to the formation of 2-, 4-, and 6-NO_2_-Trp, with 6-NO_2_-Trp being the most abundant product [21].

Protein S-nitrosylation SNO is a covalent modification of cysteine residues involving ^•^NO, peroxynitrite and its derivatives. Proteins can undergo reversible post-translational modifications for which SNO is required. Accurate prediction of SNO sites is a topic that is currently receiving much attention, because it is very important for elucidating biological changes. Several studies have shown that S-nitrosylation controls both physiological and pathological processes, including immunological response and cellular senescence [22]. Alzheimer’s disease and breast cancer are two other diseases that may be due to the abnormal S-nitrosylation of proteins [23,24].

Methionine is one of the most easily oxidized amino acids with cysteine, tyrosine, and tryptophan. Peroxynitrous acid and peroxynitrite combine with methionine to form sulfoxides, and methionine catalyses the isomerization of peroxynitrite to nitrate. The pH and methionine concentration have no effect on the distribution of nitrite, nitrate, and methionine sulfoxide, the only detectable products [25]. This is a straight bimolecular process.

There is a growing interest in the post-translational modification of proteins by methionine sulfoxide [26]. Oxidation of methionine to sulfoxide can lead to significant structural and/or functional changes in protein activity, which can be up- or down-regulated. Methionine sulfoxide has been shown to serve as a conformational transition between -helical and -sheet forms in model peptides [26], a process similar to that observed in neurodegenerative diseases.

HOCl interacts rapidly with amines and, to a lesser extent, with amides, producing chlorinated derivatives such as chloramines (R-N(R’)-Cl) and chloramides (R-C(O)-N(R’)-Cl). These compounds can be further oxidized to produce dichlorinated species (R-N-Cl_2_). The α-amino group of amino acids, the N-terminus of peptides and proteins as well as nucleophilic sites on the side of protein chains can be chlorinated (e.g., lysine, arginine). Depending on the ratio of chlorine to amino acid, chlorination of amino acids leads to the formation of organic mono- or di-chloramines. Aldehydes, nitriles, and N-chlorodimines are all likely by-products of the degradation of organic chloramines, and all three pose a risk to human health. Chloramine production interferes with protein folding and causes protein aggregation [27].

## 3. Amino Acid Nitration and Hydroxylation

### 3.1. Tyrosine Nitration and Hydroxylation

The rate constant of some reactions involved in tyrosine nitration/hydroxylation is given in Table 1, below.

**Figure 6:**
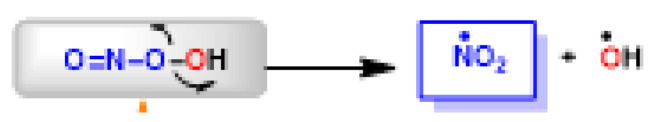

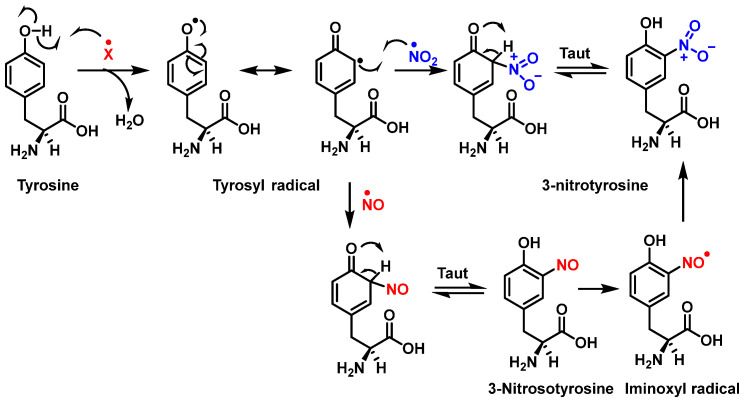
Mechanism of tyrosine nitration with either ^•^NO_2_ (in blue) or ^•^NO (in red) leading to the formation of 3-nitrotyrosine.

Figure 6 shows the mechanism of tyrosine nitration described in the literature. Because of its short half-life of 10^−2^ s, peroxynitrite cannot react directly with tyrosine residues. Instead, it decomposes into oxidizing and nitrating species, including the radicals—^•^OH and^•^NO_2_. The radical ^•^OH removes hydrogen from the phenol group of tyrosine. This produces the tyrosyl radical, which reacts with the ^•^NO_2_ to form 3-nitrotyrosine 3- NTyr [12].

The tyrosyl radical formed during the oxidation of tyrosine by various oxidizing agents reacts with ^•^NO_2_ to form 3-NTyr. It is also possible to nitrate tyrosine by an alternative route in which tyrosyl radicals react with ^•^NO to form 3-nitrosotyrosine. Using an iminoxyl radical as an intermediate, oxo-metal complexes can further oxidize this product to form NO_2_-Tyr.

**Figure 7:**
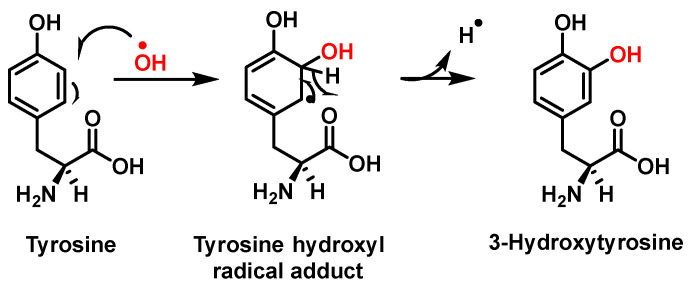
Mechanism of tyrosine hydroxylation with ^•^OH leading to the formation of hydroxytyrosine.

Hydroxylation of tyrosine follows a similar mechanism, in which ^•^NO_2_ is replaced by ^•^OH, Figure 7. In the mechanism, the ^•^OH can be added to the tyrosine phenyl ring, producing a hydroxytyrosyl radical, stable by odd-electron delocalization, to generate 3-hydroxytyrosine.

The energy values of amino acids and its derivatives have been analyzed using the zwitterion structure, as this form can be found in the cell cytosol in equilibrium with the non- zwitterion structure, as its pH is usually maintained between 7.0 and 7.4 [31].

### 3.2. Tryptophan Nitration and Hydroxylation

**Figure 8:**
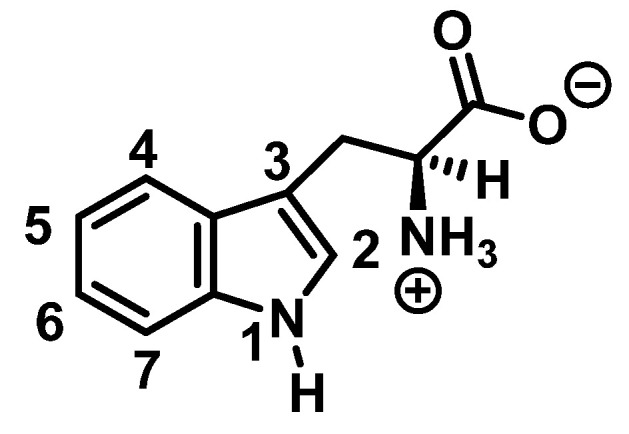
Numbering of the carbon atoms in the aromatic ring of the tryptophan molecule.

Tryptophan nitration is less frequent than tyrosine nitration. In homogenized rat brain media, treated with 1 mM peroxynitrite, 244 peptides with nitrated tyrosine were identified, compared to only 2 peptides with nitrotryptophan [32]. The numbering of the carbon atoms in the aromatic ring of the tryptophan molecule is shown in Figure 8.

Although the mechanism of the reaction between ONOO and Trp is less clear, it probably follows a similar pattern to that described for the synthesis of 3-NTyr. Tryptophanyl radicals were observed after the addition of ONOO^−^, suggesting that ONOO^−^ also modifies Trp via a radical intermediate. Unlike Tyr, whose indole can only be modified at a single carbon atom in the benzene ring, the indole of Trp has multiple reactive sites, including carbons 2-, 4-, 5-, 6- and 7 as well as nitrogen. Therefore, 1-, 2-, 3-, 4-, 5-, 6-, and 7-nitrotryptophan are all possible outcomes of the ONOO^−^ reaction with Trp. In addition, the formation of a variety of oxidation products occurs, possibly due to a tryptophanyl radical at one of these sites.

**Figure 9:**
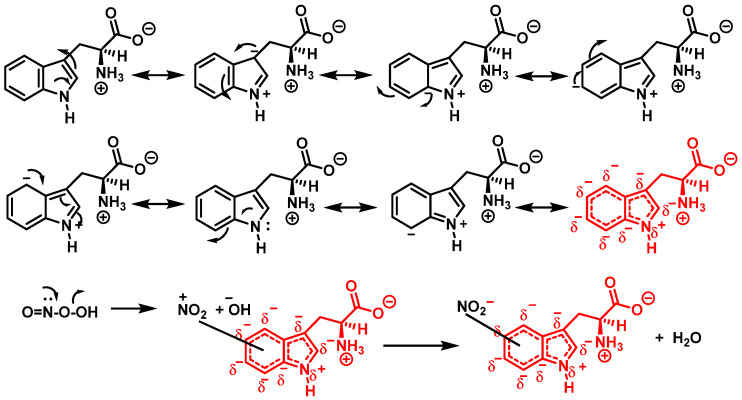
Mechanism for tryptophan nitration by addition of a nitronium ion-like species (^+^NO_2_) to the indole ring and removal of a proton in an aromatic electrophilic substitution.

In addition, Trp has been shown to nitrate in response to ONOO^−^ treatment. This occurs when ONOO^−^ combines with OH to form an ONOO radical, and subsequently decomposes to NO, which reacts with Trp. Trp, unlike Tyr residues, can react directly with ONOO^−^ to produce other oxidation products. It is possible to nitrate tryptophan by first removing a proton in an aromatic electrophilic substitution, as shown in Figure 9, and then adding a nitronium ion-like species (^+^NO_2_) to the indole ring.

**Figure 10:**
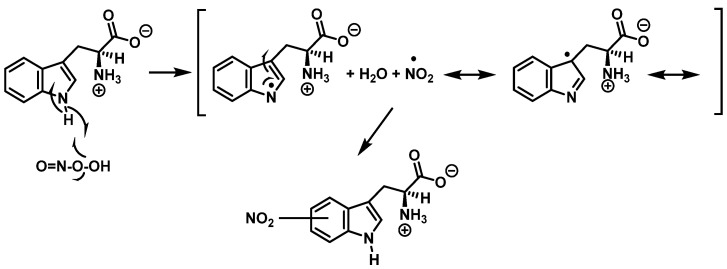
Alternative mechanism for tryptophan nitration via abstraction of the hydrogen atom from the nitrogen to give stabilized tryptophanyl radical, which can delocalize the odd electron throughout the ring, followed by addition of the ^•^NO_2_ radical.

Alternatively, nitration can be accomplished by removing the hydrogen atom from the nitrogen to produce a stabilized tryptophanyl radical capable of delocalizing the odd electron throughout the six-membered ring, followed by the addition of the nitrogen dioxide radical, either in steps or simultaneously, as shown in Figure 10. In this way, the one-electron oxidation of tryptophan is thermodynamically possible because the reduction potential E°′ (ONOO^−^,2H^+^/^•^NO_2_, H_2_O), +1.4 V [33], is higher than E°′ (tryptophanyl radical, H^+^/tryptophan), +1.0 V [34].

In summary, 6-nitrotryptophan is the major by-product of the reaction between peroxynitrite and tryptophan, followed by other nitrated and oxidized isomers such as N-formylkinurenine, and the more labile 1-nitrosotryptophan. 

**Figure 11:**
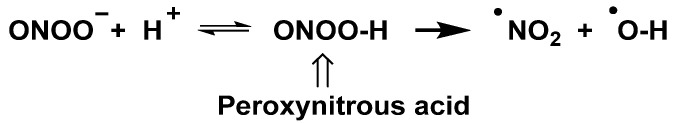
Decomposition of peroxynitrite to ^•^NO_2_ and ^•^OH.

The peroxynitrite ion is stable, but its protonated form (ONOOH, pKa = 6.5 to 6.8) decomposes rapidly through homolysis to a variety of RNS. Its decomposition can form approximately 28% free ^•^NO_2_ and ^•^OH radicals [35], Figure 11.

**Figure 12:**
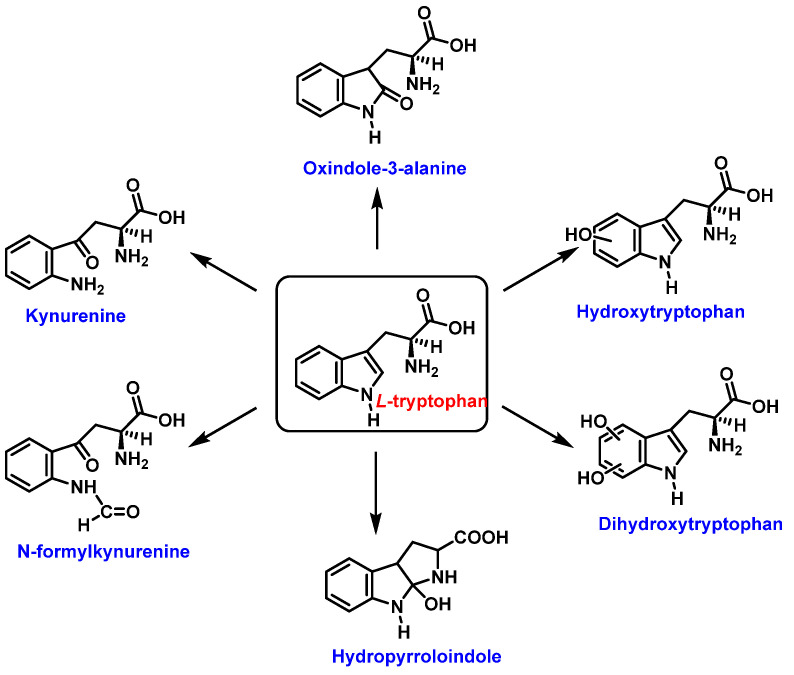
Oxidation products of L-tryptophan.

Tryptophan can be converted to 2-, 4-, 5-, 6- or 7-hydroxyderivatives, and to N-formylkinurenine and kinurenine, Figure 12.

**Figure 13:**
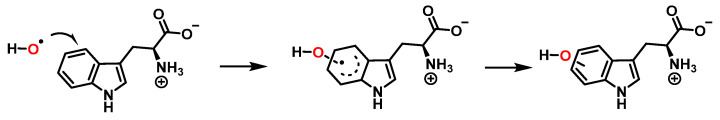
Formation of the hydroxy isomers.

Addition of the ^•^OH on the benzene ring and hydroxy isomers formation is represented in Figure 13.

**Figure 14:**
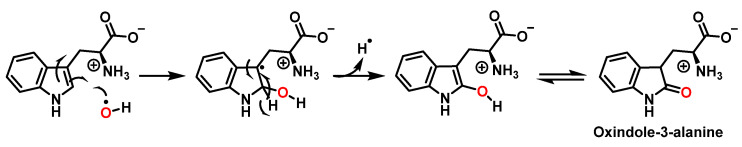
Oxindole-3-alanine formation mechanism.

For the formation of oxindole-3-alanine, there is the addition of the ^•^OH to C-2 and formation of the radical at C-3, stabilized by being delocalized with the double bonds of the benzene ring. The last step is the formation of the enol in tautomeric equilibrium with the ketone form [36], Figure 14.

**Figure 15:**
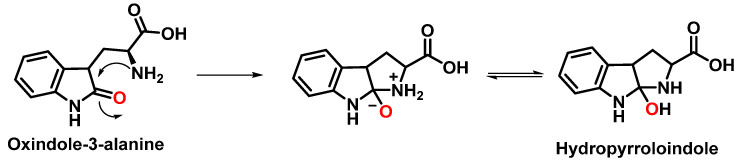
Hydropyrroloindole formation mechanism.

The formation of hydropyrroloindole from oxindole-3-alanine could be explained by the nucleophilic addition of NH_2_ to the carbonyl carbon, generating an unstable NO-acetal, Figure 15. Kato et al. (1997) found that equimolar concentrations of ONOO− and tert-butoxycarbonyl-tryptophan (Boc-Trp) produced oxindole-3-alanine (Hydropyrroloindole and N-formylkinurenine) exclusively, without identifying nitrMetation products [36].

Oxindole-3-alanine, N-formylkynurenine, and four hydroxytryptophans are formed when tryptophan undergoes a Fenton or Udenfriend reaction. According to this observation, the hydroxyl radical targets positions 2 and 3 of the pyrrole ring in addition to the aromatic nucleus. Hydroxylated derivatives are formed when the hydroxyl radical adduct is unevenly distributed with Fe-EDTA, or in a Fenton reaction. The yield of hydroxytryptophane derivatives is proportional to the concentration of Fe-EDTA up to a theoretical yield limit of 54%. The ratio of 4-, 5-, 6-, and 7-hydroxytryptophan derivatives under these circumstances is 4:2:2:3, respectively [37]. Trp subjected to the Udenfriend reaction yields approximately equal amounts of 4-, 5-, 6-, and 7-OHTrp and N-formylkynurenine.

### 3.3. Cysteine Derivatives from Reaction with Peroxynitrite

**Figure 16:**
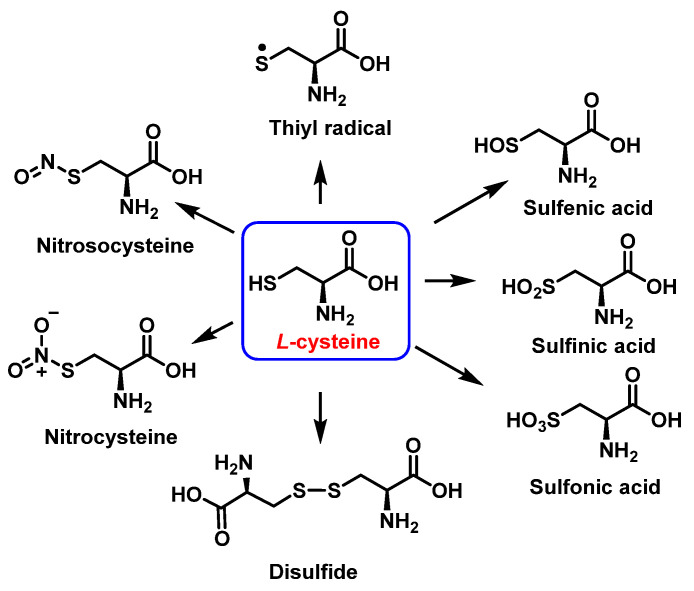
Products and intermediates detected in the reaction of cysteine with peroxynitrite.

Cysteine is the amino acid that reacts most rapidly with peroxynitrite. The products and intermediates detected in the reaction of cysteine with peroxynitrite [38,39] are specified in Figure 16.

In biochemistry, S-Nitrosylation is the covalent attachment of a nitric oxide group −NO to a cysteine thiol within a protein to form an S-nitrosothiol SNO, and has diverse regulatory roles in bacteria, yeast, plants, and in all mammalian cells [40]. It is a fundamental mechanism for cellular signaling across phylogeny, and accounts for the large part of NO bioactivity. S-nitrosylation is a precise, reversible process necessary for a wide range of cell signaling, including, for example, the red blood cell-mediated auto regulation of blood flow, essential for vertebrate life [41]. S-nitrosylation depends on enzyme activity, with three classes of S-nitrosylase enzymes, which operate in concert, analogous to ubiquitinylation [42]. The reverse effect of S-nitrosylation is denitrosylation, also controlled by enzymes. Multiple enzymes have been described and divided into two main classes that mediate denitrosylation of proteins and low molecular weight SNOs, respectively. S-Nitrosoglutathione reductase (GSNOR) is an example of the low molecular weight class [43]. These denitrosylases are involved in the removal of NO from S-nitrosylated cysteine residues, and thus can potentially remodel nitrosative stress under disorder conditions.

### 3.4. Methionine Derivatives from Reaction with Peroxynitrous Acid and Peroxynitrite

**Figure 17:**
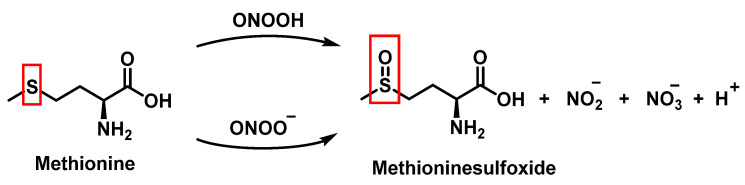
Products and intermediates detected in the reaction of methionine with peroxynitrous acid and peroxynitrite.

Peroxynitrous acid and peroxynitrite both react with methionine. The corresponding kinetic constants being K_acid_ = (1.7 ± 0.1) × 10^3^ M^−1^ s^−1^ and K_anion_ = 8.6 ± 0.2 M^−1^ s^−1^. Nitrites, nitrates, and methionine sulfoxide were the only byproducts identified, Figure 17.

For every three peroxynitrites consumed in the reaction, one nitrate and two nitrites are formed. The excess methionine concentration had little effect on the distribution of nitrite, nitrate, and methionine sulfoxide yields. pH had no effect on the production of methionine sulfoxide, nitrite, or nitrate. Methionine sulfoxide with recovered methionine corresponds to 100±4% of the methionine present at the beginning. The only methionine-derived substance identified was methionine sulfoxide [25].

## 4. Chlorination of Amino Acids

**Figure 18:**
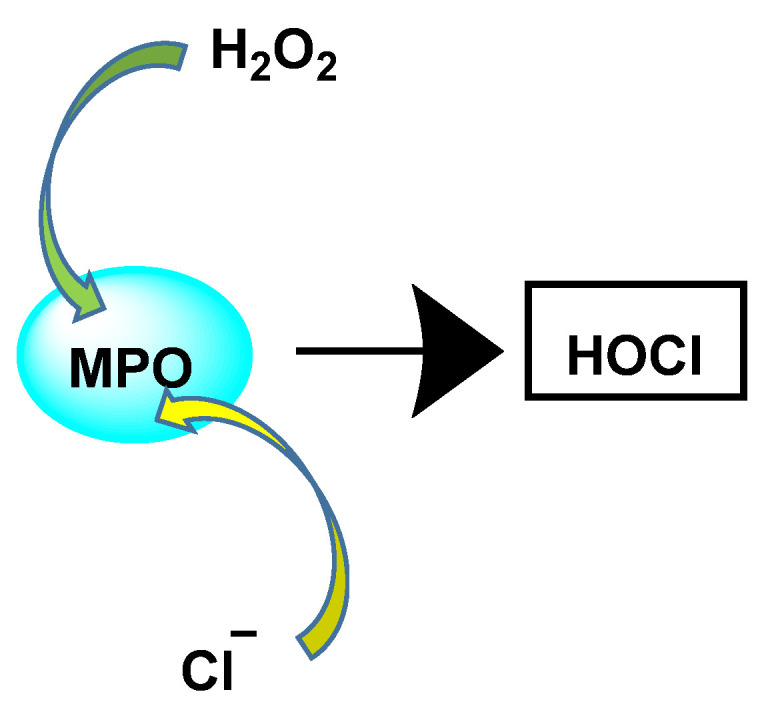
Conversion of H_2_O_2_ and chloride to HOCl.

HOCl is formed in biological systems by the enzyme heme myeloperoxidase MPO, which converts H_2_O_2_ to HOCl in the presence of chloride ion (Cl^−^) [44], Figure 18.

**Figure 19:**
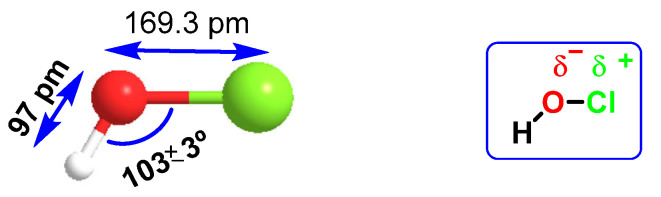
Interatomic distances and electronegativity of the HOCl molecule.

HOCl belongs to a new group of “non-antibiotic antimicrobial molecules”, known as disinfectants, which act by oxidation of organic matter. It was found in very low concentrations in the human body, synthesized by the enzyme myeloperoxidase in the cells of the immune system (neutrophils and macrophages) during an immunological process known as “respiratory burst”, and is used to fight against infections caused by bacteria. It is a chemotactic substance with excellent microbial control. The distance of the H-O bond (97 pm) is almost half that of O-Cl (169.3 pm), so there is a higher electronegativity in the HO part of the molecule, Figure 19. HOCl has a pKa of 7.5, so it coexists with ionized hypochlorite (-OCl) in solution at physiological pH. The HOCl produced has been shown to be a potent oxidant capable of chlorinating electron-rich substrates.

**Figure 20:**
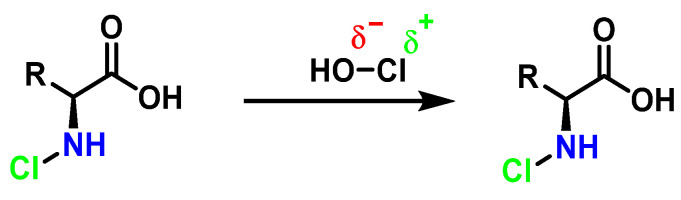
General method of chlorination of the amino group of the amino acid.

Amino acids can react with HOCl to monochloramine formation, Figure 20. Aromatic amino acids react with HOCl to form a short-lived chloro-amine, which rapidly transfers its chlorine group to another amine.

Competitive kinetic studies and stopped-flow approaches have been used to evaluate the rates of various HOCl-amino acid reactions [45]. Winterbourn used a competitive reaction with monochlorodimetone in 1985 to determine the relative reaction rates of various amino acids with HOCl. The reaction sites (amino versus side chain) were not reported, although the order of reactivity was Cys > Met > Cystine > His > Ser > Leu.

Second-order rate constants for HOCl reactions within a protein were calculated (at physiological pH, 7.4, in aqueous solution). Met > Cys > Cystine > His > Trp > Lys > Tyr > Arg > Gln > Asn [46] was the order of reactivity of the different side chains (Table 2). The constants for the reactions of HOCl with the side-chain groups of amino acids, α--amino groups, and backbone amides are shown in Table 2.

Depending on the environment, the second-order rate constant for backbone amides varies by several orders of magnitude. This is a maximum estimate based on research with cyclic dipeptides.

In view of the energy content of the chlorinated species in the amino acids studied, a little higher than the free amino acids, it can be proposed that chlorination takes place, but that the new molecule evolves by losing chlorine as a radical, or through decarboxylation of the chloramines, to form unstable imines that undergo hydrolysis and change to aldehydes, with loss of ammonia.

**Figure 21:**
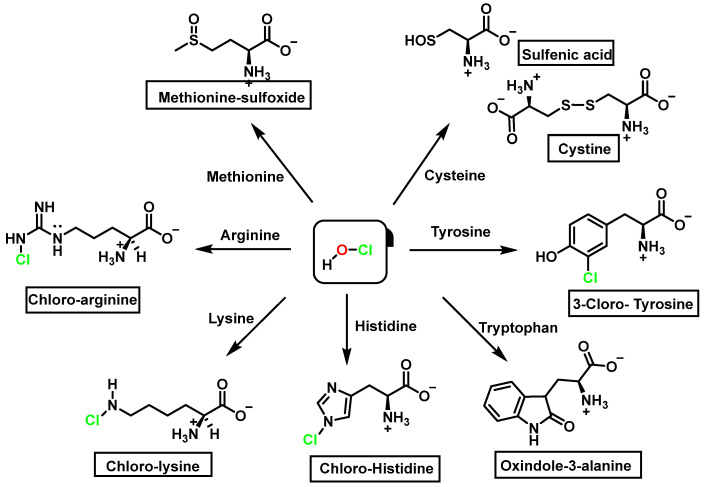
Chlorination of amino acids to a nitrogen other than the amino group adjacent to the carboxyl group.

We now consider the products and energy content of the side-chain reaction on a series of amino acids with HOCl, Figure 21.

Reaction with HOCl alters the side chains of tyrosine, tryptophan, histidine, arginine, cysteine, and methionine derivatives. The main products of HOCl-mediated Trp oxidation are 2-hydroxyindole and its tautomeric 2-oxindole derivative Trp [47]. It has also been proposed that HOCl oxidation of Trp can lead to the formation of kynurenine and N-formylkynurenine. The proposed mechanism involves HOCl reacting with the amino group to form monochloramine, which is then thermally decomposed (or catalyzed by metal ions) to form radicals. These radicals can react with additional Trp side chains to form kynurenine.

In the presence of excess HOCl, the reactions of the amine of the Lys side chain with HOCl lead to the formation of unstable mono- and dichloramines. These compounds are moderate oxidants that can transfer chlorine to other substrates while the original amine is renewed [48].

Although the His side chain reacts rapidly with HOCl to form a short-lived chloramine [49], the free amino group can also be chlorinated. At a pH of 7.4, kinetic measurements indicate that the reaction proceeds approximately the same at both sites [46]. However, because the reactivity of the imidazole ring is particularly sensitive to pH, changes in pH, an environment in which the pKa of the side chain changes, limit the rate of reaction at the side chain. This leads to a preferential reaction at the -amino group. Ring-derived chloramines have been shown to readily transfer chlorine to other amine groups, resulting in more stable chloramines. The interaction of histamine with HOCl at pH 8.0 has already been shown to occur preferentially at the (non-imidazole) amino main group [50]. 

The aromatic ring reaction of tyrosine with HOCl leads to 3-chlorotyrosine. There is no information in the literature about the by-products of HOCl-mediated oxidation of Gln and Asn side chains in aqueous solution. This is most likely due to their slow reaction rate with HOCl, which makes them very insignificant targets in proteins [46].

The following summary table lists the amino acids and their derivatives by nitration, hydroxylation, and chlorination, Table 3.

## 5. Protein Hydroxylation and Its Biological Role

Proteins are necessary for the structure, operation, and control of the body’s tissues and organs. A critical aspect of hydroxylation is the damage triggered to their structural integrity, causing loss of activity and paralysis in the regulation of metabolic pathways. 

**Figure 22:**
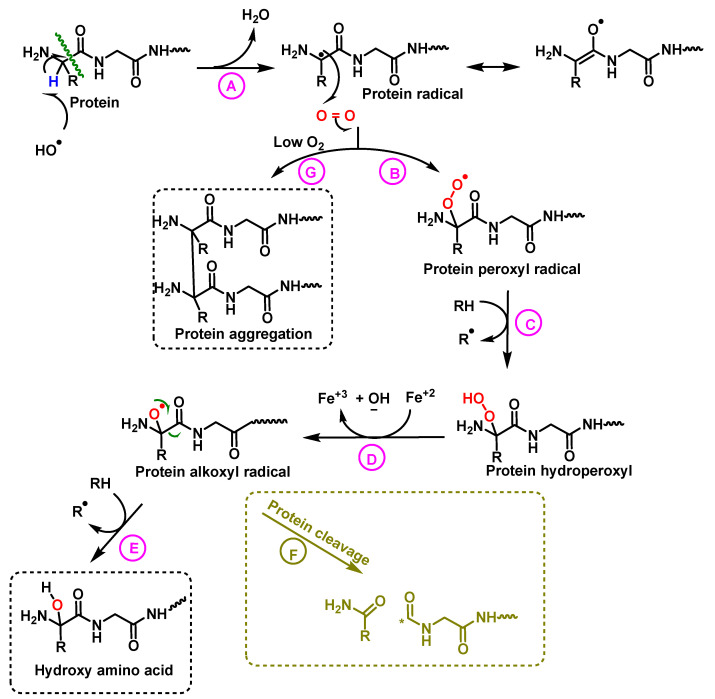
Mechanism of protein hydroxylation. The abstraction of hydrogen from the protein by the hydroxyl radical generates the alkyl radical, stabilized by resonance with the carboxyl function (**A**). The alkyl radical reacts with oxygen to form the peroxide radical (**B**). The peroxide radical abstracts another hydrogen from an adjacent protein and a hydroperoxide and an alkyl radical are formed (**C**). The hydroperoxide is reduced to an alkoxy radical in the presence of ferrous iron (**D**). Hydrogen abstraction from an adjacent protein by the alkoxyl radical forms hydroxy amino acid derivatives (**E**). In hypoxia levels the alkyl radicals form protein aggregates (**G**).

Oxidized proteins are processed by the proteasome to prevent their diffusion in the metabolic network, or their interaction with other proteins [51]. The effects of ROS on proteins and peptides are: (i) hydroxylation of residues, (ii) cleavage of peptide bonds and (iii) aggregation of proteins [1]. The nature of ^•^OH-mediated damage varies on the residue assembly in protein, Figure 22.

**Figure 23:**
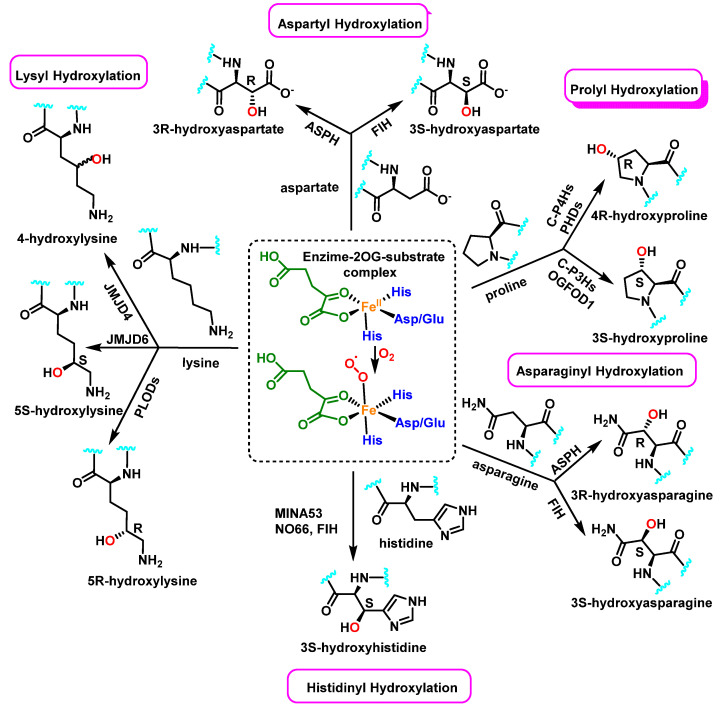
In protein substrates, the human enzyme 2OG oxygenase catalyzes the stereoselective hydroxylation of proline, lysine, asparagine, aspartate and histidine residues.

Enzymatically, protein hydroxylation is a post-translational modification catalyzed by 2-oxoglutarate-dependent dioxygenases 2-OG [52], a type of hydrolase iron-containing enzyme, and modification can take place on various amino acids, including, among others, proline, lysine, asparagine, aspartate, and histidine, Figure 23. This process was first recognized in collagen biosynthesis [53]. Some seventy 2-OG-dependent dioxygenases have been identified in the human genome and according to their functions, they can be classified into three major subclasses: histone demethylases, DNA/RNA demethylases/hydroxylases, and protein hydroxylases [54,55].

Collagen families have typical domains with a triple-helical structure shaped by three collagen α chains. Proline residues are hydroxylated by three isoenzymes of the group of collagen prolyl hydroxylases [56]. In collagens, 4-hydroxyproline (4-Hyp) residues are abundant, but some 3-hydroxyprolines also take place, and the melting temperature of collagenous triple helix is directly proportional to the 4-Hyp content; therefore, this 4-hydroxylation is critical for the stability of individual tropocollagen [57].

This type of post-translational modification affects other intracellular proteins; for example, the hypoxia-inducible factors HIFs have been found to be hydroxylated on both proline and asparagine residues [58], which affects HIF-α stability via the Von-Hippel Lindau (VHL) tumor suppressor pathway. Its hydroxylation may influence other post-translational modifications or kinase activity of the modified protein (such as Akt and DYRK1A/B), and may also alter protein-protein interactions and downstream signaling events in vivo (such as OTUB1, MAPK6 and eEF2K) [59].

The tumor protein p53 gene (Tp53), also called the guardian of the genome, is one of the most important tumor suppressors and it is mutated in more than 50% of tumors. This gene gives rise to a protein found in the nucleus of cells and plays an important role in controlling cell division and destruction. Wang et al., reported that the tumor suppressor protein p53 physically links with the Jumonji C domain-containing protein JMJD6 [60]. JMJD6 hydroxylates p53 on the lysine 382 (Lys382) residue, leading to the inhibition of its transcriptional activity. Several types of human cancers, especially colon cancer, show an up-regulation of JMJD6 expression, and this over expression positively correlates with the aggressiveness of colon adenocarcinomas. This finding suggests that JMJD6 could be considered as a therapeutic target in colon cancer [60].

## 6. Protein Nitration and S-Nitrosylation–Role in Human Diseases

**Figure 24:**
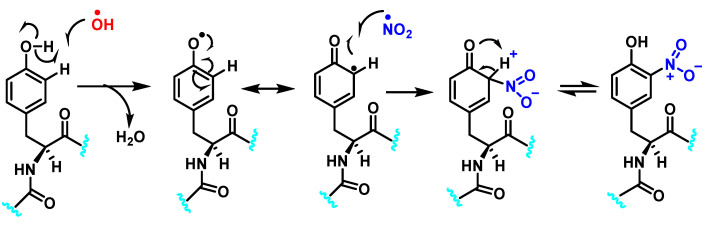
Tyrosine nitration with ^•^NO_2_ and nitrosation with ^•^NO.

Protein nitration is a post-translational modification that depends on the reactivity of the tyrosine residues present in the target protein, and can occur by reaction with peroxynitrite, by reaction of NO with protein tyrosyl radicals, by reaction of nitrite with peroxidases, or by a combination of all the above pathways [61]. Generally, it is a covalent protein modification from the addition of a nitro ^•^NO_2_ group adjacent to the hydroxyl group on the aromatic ring [12]. A stable product 3-nitrotyrosine is formed by addition of ^•^NO_2_ to the ortho position of tyrosine, Figure 24. The proximity of catalytic metal centers appears to be of critical relevance in the process of Tyr-nitration. This allows for more selectivity and, in certain cases, greater specificity in the nitration of the Tyr residue [62].

Tyr-nitration and its subsequent cascade is involved in a variety of functions, including cell signaling and the initiation and progression of disease. It has been observed in a variety of processes, including those associated with nitrosative stress such as inflammatory, neurodegenerative, and cardiovascular diseases [63]. A key role in cellular defense against oxidative stress is played by Mn superoxide dismutase MnSOD or SOD2 [64]. Several residues of the MnSOD (including Tyr34, Tyr9 and Tyr11) are susceptible to nitration, and the loss of MnSOD activity upon Tyr34 nitration implies its inactivation [65]. Some amino acids can react directly with peroxynitrite: cysteine, methionine, and tryptophan, and others do not react directly with peroxynitrite (e.g., tyrosine, phenylalanine, and histidine), but can be modified through secondary species such as hydroxyl, carbonate, and nitrogen dioxide radicals. In contrast to tyrosine, tryptophan has many sites to be nitrated [66].

Tyr-nitration is usually kept at low levels under normal physiological conditions but instead, abnormal levels of ROS and RNS appear in inflammation-associated diseases. Inflammation induced in asthma, sepsis, during transplant rejection, and in neurodegenerative diseases can be associated with an elevation in NO synthesis, which ultimately leads to increased protein nitration, especially at the tyrosine residue. This nitration leads to protein dysfunction and is implicated in pathogenesis [67]. Aulak et al., 2001, in a proteomic approach, identified more than 40 proteins that can be nitrated at the tyrosine residue and that are modified by inflammatory responses. These targets include proteins involved in oxidative stress, apoptosis, ATP production, and other metabolic functions [68].

Diabetes Type 2 DT2 is commonly associated with oxidative stress and inflammation, and endothelial dysfunction is a pivotal factor in its pathogenesis. High glucose levels stimulate the Tyr nitration in the human umbilical vein endothelial cells, inducing cell injury [69,70]. Tyr nitrated proteins in the plasma of experimental and clinical DT2 patients is higher than the control [71,72]. Serum analysis from DT2 patients shows a high level of nitration on the apolipoprotein apoA-1, and the nitration is mainly on Tyr192 [73]. Diabetic patients, compared to non-diabetic people, show a significant nitration at Tyr42 of hemoglobin [74]. Nitration of glucokinase GK (the first step of glucose metabolism, that catalyses the conversion of glucose to glucose-6-phosphate), at Tyr-289 impairs its normal expression and activity, contributing to the enzymatic changes and hepatic dysfunction of high-fat diet-induced DT2. Furthermore, nitration of GK leads to pancreatic β-cell dysfunction and apoptosis, inducing perturbation on glucose metabolism and cellular antioxidant defense mechanisms, and finally increasing susceptibility to insulin resistance and DT2 [75].

Several pathologies affecting the cardiovascular system are associated with increased production of nitric oxide and/or peroxynitrite-derived oxidants, mainly due to a decrease in antioxidant detoxification routes [5]. In cells, Tyr-nitration promotes faster clearance or the storing of derivative proteins. Immunological identification of tyrosine nitration and subsequent functional assays revealed that protein nitration may be involved in a variety of functions, possibly including cell signaling and disease initiation and progression [76].

The main nitrated proteins in atherosclerosis are apoA-1, apoB-100, and fibrinogen [77]. Tyr nitration of prostacyclin synthase PCS (this enzyme belongs to the family of cytochrome P450 isomerases, with dilatory functions in the normal vasculature), is associated with the development of atherosclerosis in patients with diabetes [78]. Heavy nitration of PCS from atherosclerotic vessels is found in comparison with that from normal tissue, and its inactivation by Tyr nitration favors atherosclerotic processes [79].

Alpha-enolase (a glycolytic enzyme the catalyzes the conversion of 2-phosphoglycerate to phosphoenolpyruvate, expressed in adult human tissues, including the liver, brain, kidney, and spleen), is a main target for nitrative derivatives in diabetic patients. Nitration of α-enolase reveals a significant contribution to its inactivation. Tyr 257 and Tyr 131 of α-enolase are the most susceptible residues to nitration in diabetic rat hearts [80]. Nitration of myofibrillar creatine kinase MM-CK and its inhibition are involved in heart failure in vivo [81].

Oxidative injuries such as nitrosative stress have been implicated in serious neurodegenerative disorders, including Alzheimer and Parkinson disease (AD and PD) and Amyotrophic Lateral Sclerosis ALS.

Sultana et al., 2006, using a proteomic methodology, identified enolase, glyceraldehyde-3-phosphate dehydrogenase, ATP synthase alpha chain, carbonic anhydrase-II, and voltage-dependent anion channel-protein as the targets of nitration in AD hippocampus, a region that shows a extensive accumulation of amyloid beta-peptide, compared with the age-matched control brains [82]. In 2011, Kummer et al., recognized the peptide amyloid β (Aβ) as an ^•^NO target, which is nitrated at tyrosine residue 10. Nitration of Aβ accelerated its aggregation, and was detected in the core of Aβ plaques of APP/PS1 mice and AD brains. NOS2 (inducible NOS, iNOS) deficiency or oral treatment with the NOS2 inhibitor L-NIL strongly decreased 3NTyr(10)-Aβ, overall Aβ deposition and cognitive dysfunction in APP/PS1 mice. Further, injection of 3NTyr(10)-Aβ into the brain of young APP/PS1 mice induced β-amyloidosis. This suggests a disease modifying role for NOS2 in AD, and therefore represents a potential therapeutic target [83].

Parkinson’s disease (PD) is marked by a selective deterioration of dopaminergic neurons in the brain stem and it is the second most widespread neurodegenerative disorder. The pathogenic process of PD is not completely known, but it is believed to be involved with the imbalance of nitric oxide ^•^NO. Recent studies have suggested that ^•^NO, through the modification of protein’s cysteine residues can contribute to the pathogenesis of PD [84]. Stykel and Ryan, 2022, provide a summary of how RNS stores in PD, considering several sources of RNS (nNOS, iNOS, nitrate, and nitrite reduction), and describe evidence that these sources are up-regulated in PD. They document that over 1/3 of the proteins deposited in Lewy Bodies are nitrosylated by RNS and provide a broad description of the deleterious effects in neurons, identifying specific nitrated proteins in neurons that are implicated in PD pathogenesis with an emphasis on exacerbation of synucleinopathy. They outlined the fact that nitration of alpha-synuclein (aSyn) leads to aSyn misfolding and toxicity in PD models and, furthermore, delineating how RNS modulates known PD-related phenotypes including axo-dendritic, mitochondrial, and dopamine dysfunctions [85].

ROS/RNS species and mitochondrial dysfunction are a hallmark of amyotrophic lateral sclerosis ALS, a fatal progressive neurodegenerative disease, also known as Lou Gehrig’s disease. The most important degeneration of neuronal cells occurs in the motor neurons of the spinal cord, brainstem, and brain and begins in a focal manner in the central nervous system, and progressively spreads inexorably [86]. The disease has a heterogeneous course in all patients, but most of them die of respiratory muscle weakness within 3-5 years of onset. Like other neurodegenerative diseases, ALS has genetic, metabolic, and environmental triggers [87]. So far, there is no cure available for this disease, and treatment focuses on a combination of neuroprotective medication, multidisciplinary clinics, and respiratory support. Altered neuronal function is associated with ROS/RNS stress and is reflected in changes in certain blood levels metabolites, which can be used as biomarkers of tissue function and thus of disease progression and/or patient response to treatment [88,89]. Several ALS-related pathogenic mechanisms involve redox-sensitive proteins, such as disulphide isomerase PDI, thioredoxin and glutathione GSH [90,91,92]. Recent evidence implies that redox homeostasis is a central and primary mechanism in ALS and may be of greater importance than previously attributed [93]. Dysregulation of NADPH oxidases NOx family enzymes are implicated in ALS. NOx generates superoxide ^•^O_2_^−^ and controls the production of pro-inflammatory interleukin IL-1β and the factor TNF-α, which are elevated in the plasma of ALS patients [94,95] and the overexpression of SOD1 in spinal motor neurons [96].

Immune responses can be affected by air pollutants, including combustion-generated particulate matter, unburned semi-volatile hydrocarbons, and exhaust gases [97]. These components induce inflammatory responses in the airways, enhance allergen responses, and reduce resistance to bacterial and viral infections [98,99,100,101]. Post-translational modifications, including nitration, glycosylation, phosphorylation, and cysteinylation, among others, can affect the immunogenicity of proteins and play a role in the triggering of autoimmune responses [102]. Changing a single amino acid residue in allergens can alter their allergenicity, as illustrated by point mutations in Bet v1a, which drastically affect immunoglobulin (Ig)E binding [103,104]. Similarly, nitration of tyrosine residues can alter the immunogenicity and allergenicity of proteins [105], potentially modifying immune responses [106].

The prevention of protein nitration in vivo implies a correct balance between the generation of reactive oxygen and nitrogen species and their detoxification by the cellular antioxidant system (glutathione, superoxide dismutase enzymes, catalase... [107]). Tyr nitration of proteins also occurs under physiological conditions, but is markedly increased in the pathological state. From this point of view, it is desirable to develop compounds that can decompose and catalytically remove peroxynitrite, and reactive RNS/ROS species as therapeutic agents [108,109], or to prevent their formation through dietary intake of antioxidant compounds, such as some vitamins and polyphenols, especially flavonoids [110], present in many fruits and vegetables. Flavonoids are naturally occurring antioxidants and are abundant in vegetable foods, and have also received tremendous attention for their efficiency in Tyr nitration inhibition, but they also have other significant health benefits [111,112].

S-nitrosylation is a post-translational modification that regulates protein function through the reaction of NO with a cysteine thiol group on target proteins [113]. In the physiological state, S-nitrosylation is an important modulator of signal transduction pathways, similar to phosphorylation [114]. However, due to aging or RNS proliferation, excess NO is generated, and together with tyrosine nitration, aberrant S-nitrosylation reactions can occur, affecting protein misfolding, mitochondrial fragmentation, synaptic function, apoptosis, or autophagy [115]. Thousands of proteins with potential sites for S-nitrosylation have been identified. Although most cellular proteins possess multiple cysteine residues, only some cysteine residues are S-nitrosylated, one of the determinants being proximity [116]. Another involves the presence of a characteristic SNO motif with certain amino acid residues adjacent to the target cysteine [117]. Local hydrophobicity can promote the specificity of S-nitrosylation by providing increased stability of the S-nitrosothiol group [118]. 

S-Nitrosylation leads to conformational changes in protein structure, resulting in the derivatization of sulphenic acid (-SOH), sulfinic acid (-SO2H) or sulphonic acid (-SO3H) from the thiol group of cysteine. Sulfonation (-SO3H) cannot be reversed by known enzymes, and will therefore result in permanent changes in protein structure and activity [119]. On two neighboring cysteine residues, S-nitrosylation of one of them can facilitate the formation of disulphide bonds between them [120]. Conversely, if both cysteine residues are nitrosylated under severe nitrosative conditions, S-nitrosylation inhibits the formation of disulphide bridges [121].

S-nitrosylation can trigger conformational changes, activating or inhibiting protein activity, altering protein-protein interactions, protein aggregation, or influencing protein localization. Abnormal S-nitrosylation is connected to various human diseases, such as diabetes, heart failure, asthma, and pulmonary hypertension.

These alterations affect cellular signal transduction pathways and neuronal function [122]. Under pathological conditions, aberrant S-nitrosylation stimulates cell destruction processes and thus contributes to neurodegeneration. S-nitrosylation-mediated modifications include protein misfolding, endoplasmic reticulum (ER) stress, mitochondrial dysfunction, synaptic degeneration, and ultimately apoptosis [123]. Nitrosative stress is implicated in several neurological disorders, such as acute hypoxia-ischemia and chronic neurodegenerative diseases. Pharmacological inhibition of nNOS or deletion of the gene encoding nNOS provides neuroprotection against ischaemia [124].

Proteins with aberrant S-nitrosylation (so-called SNO proteins) play a crucial role in the pathogenesis of neurodegenerative diseases, including Alzheimer’s and Parkinson’s diseases. S-nitrosylation at the thiol group of cysteine is a redox reaction, chemically different from the Tyr nitration, representing another NO-dependent post-translational modification generated by the reaction of tyrosine with peroxynitrite [125].

PD is induced either by genetic factors (which appear in the early development of the disease), or by environmental factors, possibly including agricultural insecticides, herbicides, fungicides, or other neurotoxins that act as mitochondrial toxins and thus generate oxidative and nitrosative stress [126]. S-nitrosylation of proteins, including parkin, DJ-1, X-linked inhibitor of apoptosis protein (XIAP or IAP3), peroxiredoxin (Prx) 2, and glyceraldehyde-3-phosphate dehydrogenase (GAPDH), is involved in the pathological activity, and can influence to ubiquitin-proteasome loss. 

Parkin is an E3 ubiquitin ligase involved, via the ubiquitin-proteasome disposition (UPS), in the control of protein degradation [127]. Parkin is also involved in protein degradation during ER stress. It interacts with chaperones, heat shock protein (Hsp)70 and the carboxyl-terminal protein, which interferes with Hsp70 (CHIP), and thus participates in ER-associated degradation ERAD [128]. Loss of parkin activity negatively interferes with protein degradation, leading to aggregation of neurotoxic proteins and consequent ER stress [129]. Parkin also silences the transcription of the oncogene p53 and aids a neuroprotective role against PD-related apoptosis of dopaminergic neurons [130]. Mutations in parkin PARK2 have been acknowledged as instrumental in early onset PD [131]. Levels of SNO-parkin are notably up-regulated in the brains of humans with sporadic PD, and in animal models of PD, reinforcing the role for S-nitrosylated parkin in disease pathogenesis [132].

Insulin, released by the β-cells of the pancreatic islets in response to an increase in blood glucose levels, is a critical regulator of metabolism. It triggers the uptake of glucose and fatty acids in liver, adipose tissue and muscle, and enhances the storage of these substances as glycogen and lipids. Down regulation of insulin synthesis, secretion, haulage, deprivation or signal transduction initiates failures in nutrient uptake and storage, leading to types 1 and 2 diabetes and metabolic dysfunction. Insulin signaling is intimately linked to protein S-nitrosylation [133]. Prepro-insulin production and insulin maturation by proteolytic processing in β-cells occur inside the ER/Golgi, and mature insulin is stored in insulin-secretion granules ISG, within β-cells, awaiting signals for secretion. In islets, NO is mainly generated by nNOS, and may act as a mediator or inhibitor of glucose-stimulated insulin secretion GSIS [134]. The complex roles of NO in GSIS can be explained by S-nitrosylation of different target proteins, activating or inhibiting the insulin releasing [135].

## 7. Protein Chlorination and Its Role in Ageing and Human Diseases

Reactions of HOCl with amino acids can also occur in proteins and peptides. Amino acid residues present in proteins react with HOCl due to its high reactivity [136].

**Figure 25:**
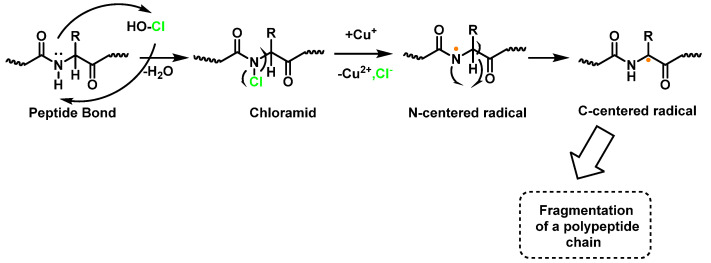
Formation of chloramide.

Within proteins, the sulphur of cysteine Cys and methionine Met react rapidly with HOCl, but also the side chains of lysine (Lys), histidine (His), tryptophan (Trp), tyrosine (Tyr), and the -amino groups are oxidized and/or chlorinated, but the reactivity of the functional groups in amino acid side chains can vary significantly. The treatment of small globular proteins, such as insulin (5.7 kDa) and lysozyme (14.4 kDa), with increasing concentrations of HOCl, leads to the modification of several amino acid residues (His, Lys, Arg, Tyr, etc.). The result of the HOCl-protein interaction is fragmentation, due to the cleavage of a peptide bond [137]. When HOCl interacts with a peptide bond, the chloramide is formed, but the reaction rate with the amide of the peptide bond is much slower than with an amino group. The reaction rate constant depends, to a large extent, on the chemical structure of the compound. In aqueous media, chloramines hydrolysis slowly with peptide bond breakage and protein fragmentation. In addition, the *N*–Cl bond can undergo homolytic cleavage with the formation of an *N*-centred radical, e.g., in the presence of transition metal ions [138]. The *N*-centred radical is short-lived, these species undergo rapid migration reactions of 1,2 hydrogen atoms to give α-carbon centred radicals stabilized by C=O conjugation with most peptides, leading to the fragmentation of the polypeptide chain, as shown in Figure 25.

**Figure 26:**
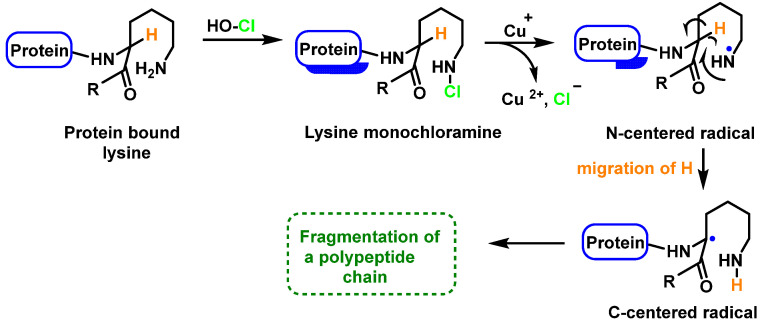
Formation of the radical centered on the N of Lys with subsequent transformation to a C-centered radical.

Peptide bond cleavage can also occur by the formation of chloramide or chloramine in the reaction of HOCl with the amide group of the glutamine side chain, or with the amino group of lysine, with an intramolecular rearrangement via a cyclic six-bond intermediate, transformed into a radical centered on the C atom of the peptide bond. Under aerobic conditions, this C-centered radical is rapidly converted to a peroxyl radical with subsequent degradation of the polypeptide chain, Figure 26.

During aging, the dynamic regulation of a balanced and functional proteome is progressively modified [139], leading to the accumulation of denatured, aggregated, or oxidized proteins, causing cellular damage and tissue deterioration [140]. The proteasome is the cellular proteolytic system responsible for the removal of non-functional or excessive proteins and plays a key role in aging [141]. Senescent cells have elevated levels of modified proteins, so their expression and function are negatively affected during aging. Their dysfunction is due not only to reduced expression of proteasome subunits and altered assembly of protein complexes, but also to modified proteins that are unable to perform their function. Reduced proteasome activity during aging has been detected in numerous human and mammalian tissues and organs (skin, lymphocytes, heart, muscle, spine, brain, liver, retina, adipose tissue, etc.) [142].

Genetic manipulation is not feasible for proteasome activation, so clinical effort has focused on identifying natural or synthetic proteasome activators with antioxidant and anti-aging properties. Among these compounds are pollen, oleuropein, curcumin, and the synthetic peptide PAP1 (Proteasome Activating Peptide-1) [143,144,145]. The transcription nuclear factor (erythroid-derived 2)-like 2 Nrf2 is known to induce the expression of antioxidant enzymes, including proteasomal subunits, so it is of clinical interest to ensure its activation [146]. Another compound that activates Nrf2 is 18α-glycyrrhetinic acid (18α-GA), which in turn induces proteasome function and increases lifespan of human fibroblasts [147]. The Nrf2/ARE pathway (antioxidant response element) is an important cell signaling mechanism in maintaining redox homeostasis in humans [148]. Dietary flavonoids, as luteolin, apigenin, quercetin, myricetin, rutin, naringenin, epicatechin, and genistein activate the Nrf2/ARE pathway in both normal and cancer cells [149].

Chlorinative stress influences the pathogenesis of neurodegenerative diseases [150]. In the brain, chloride ions are present at the concentration of 0.01–0.1 M [151]. HOCl can be generated by activation of microglia and secretion of myeloperoxidase, or by the macrophage infiltration and neuronal expression of myeloperoxidase [152,153]. Numerous articles have verified the toxicity of HOCl in central nervous system tissue [154], reporting that MPO was expressed in brain tissue of AD affected patients, and 3-chlorotyrosine was detected as a biomarker of HOCl production in AD hippocampal proteins with its level in diseased brain samples being three times higher compared with control samples [153]. Halogenation has a clear effect on the self-assembly of β-amyloid peptide derivatives [155].

## 8. Conclusions

Free radicals play a dual role: as a physiological balance. they are beneficial compounds involved in cell signaling, but at the pathological level they are toxic when their production is triggered in an uncontrolled manner. Thus, they play a dual role in cell metabolism. When an overload of free radicals cannot be processed by the antioxidant mechanism (enzymes or peptides such as glutathione or superoxide dismutase SOD, etc.), their accumulation in the body generates a phenomenon called reactive stress. This process occurs asymptomatically and plays an important role in the development of chronic and degenerative diseases such as cancer, autoimmune disorders, aging, cardiovascular, and neurodegenerative diseases. So, identifying the molecular changes in peptides and proteins caused by free radicals is an important challenge to understand, prevent, and try to treat these diseases. The role that antioxidants (tocopherol, polyphenols, flavonoids…) can play in supporting antioxidant defenses is well known and has been addressed in hundreds of scientific publications over the past years.

In this study, the focus of the research has been on two complementary aspects: (i) on analyzing the capacity of some amino acids to react with peroxynitrite and HOCl, and the free radicals ^•^OH, ^•^NO_2_, so that in the process of nitration, hydroxylation or chlorination, the peptides and proteins may lose their physiological function. At the same time, a multilevel biological mechanism was provided to explain this property of the compounds studied; and (ii) on the potential energy associated with the original molecules and the compounds produced, as determined by molecular mechanics. Following these chemical considerations, we have examined four amino acids that are susceptible to hydroxylation and nitration. Therefore, the derived compounds cannot be used by cellular metabolism to form part of proteins or, when these radicals attack peptides and proteins, they may cause their loss of function.

We hope that this review will help to assess the potential damage that reactive species can cause to free amino acids and their corresponding residues in peptides and proteins.

## Figures and Tables

**Table 1:** Chemical reactions involved in the nitration/hydroxylation of tyrosine.

Reaction	k (M^−1^ s^−1^)	Reference
*Tyr + ^•^OH → Tyr(OH)^•^*	1.2 × 10^10^	[28]
*2 Tyr(OH)^•^ → Tyr-OH + products*	3.0 × 10^8^	[28]
*Tyr^•^ + ^•^NO_2_ → NO_2_Tyr*	3.0 × 10^9^	[29]
*Tyr^•^ + ^•^NO → NOTyr*	1.0 × 10^9^	[30]

**Table 2:** Constants determined for HOCl reactions with amino acid side-chain groups.

Residue	K_2_ M^−1^ s^−1^	Residue	K_2_ M^−1^ s^−1^
Cystine	1.6 × 10^5^	Arginine	26
Cysteine	3.0 × 10^7^	Tyrosine	44
Histidine	1.0 × 10^5^	Backbone amides	<10
Methionine	3.8 × 10^7^	Lysine	5.0
Tryptophan	1.1 × 10^4^	Glutamine	0.03
α-amino	1.0 × 10^5^	Asparagine	0.03

**Table 3:** Amino acid derivatives from nitration, hydroxylation and chlorination.

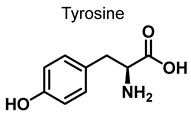	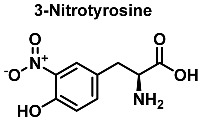 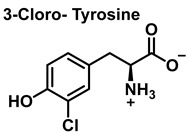	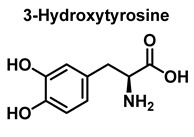
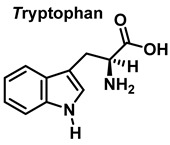	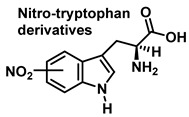 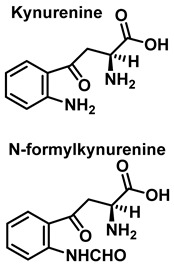	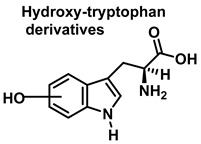 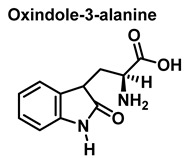
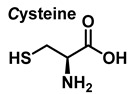	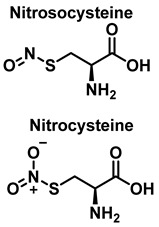 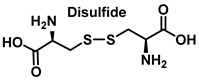	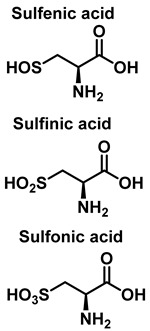
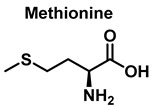	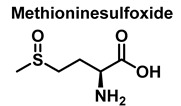	
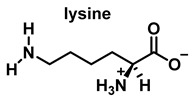	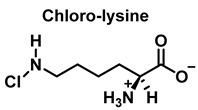	
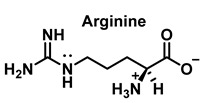	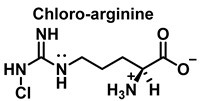	
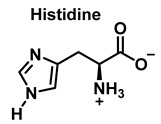	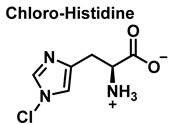	

## Data Availability

Not applicable.
